# Social franchising of community-based HIV counselling and testing services to increase HIV testing and linkage to care in Tshwane, South Africa: study protocol for a non-randomised implementation trial

**DOI:** 10.1186/s12889-020-8231-x

**Published:** 2020-01-29

**Authors:** Simukai Shamu, Charles Chasela, Jean Slabbert, Thato Farirai, Geoffrey Guloba, Nkhensani Nkhwashu

**Affiliations:** 1 Foundation for Professional Development, Health Systems Strengthening Division, Pretoria, South Africa; 2 University of the Witwatersrand, School of Public Health, Johannesburg, South Africa; 3 University of the Witwatersrand, Department of Epidemiology and Biostatistics, School of Public Health, Johannesburg, South Africa; 4 Right to Care, EQUIP, Pretoria, South Africa

**Keywords:** Social franchise, HIV testing, HIV positivity, Linkage to care, HIV testing and linkage cost, South Africa

## Abstract

**Background:**

Meeting the ambitious UN 90–90-90 HIV testing, treatment and viral load suppression targets requires innovative strategies and approaches in Sub-Saharan Africa. To date no known interventions have been tested with community health workers (counsellors) as social franchisees or owner-managed businesses in Community-based HIV counselling and testing (CBCT) work. The aim of this methods paper is to describe a Social franchise (SF) CBCT implementation trial to increase HIV testing and linkage to care for individuals at community levels in comparison with an existing CBCT programme methods.

**Methods/design:**

This is a two arm non-randomised community implementation trial with a once off round of post-test follow-up per HIV positive participant to assess linkage to care in low income communities. The intervention arm is a social franchise CBCT in which unemployed, self-employed or employed community members are recruited, contracted and incentivised to test at least 100 people per month, identifying at least 5 HIV positive tests and linking to care at least 4 of them. Social franchisees receive approximately $3.20 per HIV test and $8 per client linked to care. In the control arm, full-time employed HIV counsellors conduct CBCT on a fixed monthly salary. Primary study outcomes are HIV testing uptake rate, HIV positivity, Linkage to care and treatment rate and average counsellors’ remuneration cost. Data collection will be conducted using both paper-based and electronic data applications by CBCT or SF counsellors. Data analysis will compare proportions of HIV testing, positivity, linkage to HIV care and treatment rates and counsellors’ cost in the two study arms.

**Discussion:**

The study will provide important insight into whether the SF-delivered CBCT programme increases testing coverage and linkage to care as well as reducing CBCT cost per HIV test and per HIV positive person linked to care.

**Trial registration:**

Pan African Clinical Trial Registry PACTR201809873079121. The trial was retrospectively registered on 11 September 2018.

## Background

South Africa has consistently remained the country with the largest number (7.9 million) of people living with HIV globally. More than 1 in five (20.6%) adults (15–49 year olds) live with HIV [1]. An incidence of 1.00 has been reported among the 15–24-year-old people of which women have disproportionately higher incidence (1.51) than men (0.49). This incidence translates to 200,000 adults infected with HIV annually as at 2017 [1]. The UNAIDS estimate that 86% of the people living with HIV in South Africa know their HIV status and only 56% are on treatment while less than half (45%) are virally supressed [2]. Only 29% of men aged 15–49 and 17% of women have never tested and 59% of women and 45% of men tested and received results in the last 12 months [3]. Although HIV testing rates have increased over the last decade, low levels of testing, treatment and viral suppression in an HIV epidemic country need urgent improvement to meet the UN’s 90–90-90 targets. The pandemic varies by place in South Africa with as high as 27% prevalence in KwaZulu-Natal province and as low as 12.6% in the Western Cape Province while Gauteng province, where our proposed trial will be conducted, has a 17.6% HIV prevalence. Gauteng province has the second lowest rates of HIV testing for women (54%) and third lowest for men (41%) in South Africa [3]. The province accounts for a third (34.2%) of all annual HIV deaths in the country and lower (75%) than national average (86%) proportion of people who know their HIV status [4]. Only 55% are on treatment and 46.4% are virally suppressed. Without new or strengthened HIV prevention interventions the province will not meet the three UN HIV targets for testing, treatment and viral suppression [4].

The Department of Health (DoH) through its national, provincial and district structures provides a framework for and direct the provision of HIV testing services (HTS) to all people in South Africa through health facilities (private and public). HTS are provided through health facilities and community-based services. In the latter, clients are reached in homes, workplaces and public community places such as educational institutions. Since the advent of antiretroviral therapy, HIV testing was initially solely conducted by clinicians and nurses in health facilities. Facility-based HIV testing however suffered from enormous individual, relationship, community and health system barriers leading to low levels of HIV testing uptake and treatment including missing people at high risk and delaying treatment [5–7]. To address this, community-based HIV counselling and testing (CBCT) approaches were introduced that saw an increase in HIV testing and ART uptake in South Africa [8–10]. See Table 1 for definitions of terms used in this paper. Also, to increase the number of people in the HIV testing and treatment, clinicians and nurses were replaced by lay counsellors. CBCT in South Africa relies on a cadre of workers, loosely termed counsellors, mostly employed by or volunteering with non-governmental organisations. These cadres, coined community health workers (CHWs), numbered 40,000 in South Africa in 2004, almost equal in number to the nurses in public health facilities (43,000) [11], and rose to 73,000 in 2011 [12, 13] In 2006, the WHO introduced a quick win strategy for HIV care work by task shifting HIV work to CHWs. To date, studies have shown the success of this cadre in CBCT including increased rates of HIV testing uptake, reaching people with high CD4 count, reaching first time testers and linking clients to care [14–20]. CHWs have been described as the backbone of workers in HIV testing [21, 22]. One of the challenges facing CBCT programmes include the absence and inadequate health information infrastructure to enhance clients’ (re) linkage to care and support [23]. Due to below optimum levels of testing and treatment, South Africa still requires more innovations to achieve universal test and treat goals enshrined in the 90–90-90 UN targets adopted by South Africa, despite implementing CBCT with lay councillors over the last decade.

**Table 1:** Definitions of terms used in the study

Terms	Description
Community-based HIV counselling and testing (CBCT)	HIV testing services offered outside of the health facilities but in the community where people live or work. HIV testing services include a general package that includes mobilising clients for testing, consenting, testing, counselling and disseminating HIV prevention services and relevant referrals
Community health workers (CHWs)	Members of the community selected, employed or volunteering to provide basic preventive, promotional and rehabilitative health and medical care to their community members
Health facility-based HIV counselling and testing	HIV testing services offered at the health facilities including consenting, testing, counselling and disseminating HIV prevention services and relevant referrals. Health facilities refers to public and private health clinics. They offer services defined below under HIV testing services.
HIV testing services	These include pre-counselling, testing, post-counselling, condom distribution, sexual behaviour change and communication, referral for care and treatment including medical male circumcision, pre-exposure prophylaxis, HIV treatment. These will be customised per participant’s situation including test result.
Key population	Groups of people defined and known to be of greater risk of HIV infection than the average members of the community. These include MSM, FSWs, people who inject drugs (PWID), transgender people and People in Prisons or other enclosed settings.
Mobile Application	A mobile device such as a tablet loaded with application software that includes the use of GPS triangulation and a questionnaire to collect, save and transmit respondents’ data to enable real-time reporting and informed decision-making
Participant mobilisation	Marketing and informing clients about HIV testing services to create demand for HIV testing services. Mobilisation will be conducted by CBCT team members before and on the day of testing in each community where HIV testing is conducted. Mobilisation will include promotion and awareness campaigns using loud inhalers, distributing pamphlets, erecting banners and talking to passers-by about HIV testing services offered and inviting them to test for HIV.
Social Franchise (SF)	“a network of private individuals/ providers that are linked through agreements to provide socially beneficial health services under a common franchise brand”. A ‘franchisor’ manages the brand and oversees the administration of the program while a “franchisee” implements the brand according to laid down rules
Systematic Home-Based testing and counselling	Mapping Households at the community level and offering door-to-door HIV testing services to household members in the target community. The process was done systematically through visiting all houses, one after the other, in a community in order to avoid missing any dwelling.

Social franchising of health services has increasingly been adopted by the health sector from the commercial industry to address the universal access to health for all by 2015. The concept of social franchising aims to increase coverage, affordability and effectiveness of services by using a commercial relationship between the franchisor and franchisee [24, 25] The franchisor, who owns the brand, defines a product and brand, delivery mode, quality assurance, quality standards and provides a brand and staff training while a franchisee implements the product work under the franchisor’s guidance and brand name and product [26]. To date no known interventions have been tested with community health workers (counsellors) as social franchisees or owner-managed businesses in CBCT work. CBCT requires working with people who live and work in the community, people with knowledge of the community hotspots and requires empowering these cadres to prepare them for sustainable CBCT work once the NGO funding ends. To this effect, the Foundation for Professional Development (FPD) designed a brand called Social franchise community-based HIV counselling and testing (SF CBCT) in 2016 for piloting in comparison with the generic FPD CBCT programme [27] to increase HIV testing and treatment opportunities for individuals at community level. In this paper, we present the methodology for a SF CBCT trial within an existing FPD CBCT programme. The methodology allows for testing for differences in the use of the mobile electronic data application system to capture client data for linkage to HIV care and treatment purposes as implemented by both FPD and SF counsellors additionally.

## Methods

### Design

The study is a two arm non-randomised community implementation trial with a once off round of post-test follow-up per HIV positive participant to assess linkage to care. Clients will be tested by one of the HIV testing strategies – either by an existing FPD counsellor or a SF HIV counsellor under the sub-contracted model. Each study arm has 19 counsellors conducting CBCT. The overall study design is illustrated on Table 2.

**Table 2:** Process flow for the FPD and Social Franchise HTS

Item	Activity	FPD	Social franchise
1. Counsellor Recruitment and contracting	Contract	1. Employed by FPD 2. Normal employment contract 3. Normal payroll contract 4. Work for 8 h a day	1. Unemployed, self-employed or employed elsewhere, not employed by FPD 2. Contracted by franchisee-franchisor contract 3. No restriction on hours, days worked 4. Test >/= 100 people per month 5. Test>/5 HIV + tests per month 6. Link >/= 4 HIV + clients per month
	Remuneration	1. Fixed monthly salary	1. Paid $3.20 per HIV test 2. Paid $8 per HIV + client linked
	Training	Training focussed on: 1. Pre-test, test, post-test, documentation, referral and linkage to care	Training focussed on: 1. Pre-test, test, post-test, documentation, referral and linkage to care 2. business development & management
2. Mobilisation	Community level mobilisation	Community events; flyers; campaigns (e.g. couples counselling); conducting systematic door-to-door mobilization campaigns distributing flyers, and condoms	Community events; flyers; campaigns (e.g. couples counselling); conducting systematic door-to-door mobilization campaigns distributing flyers, and condoms
	Individual level mobilisation	Informing households on the benefits and availability of testing for HIV; scheduling household level testing appointments and/or actively recruiting clients for same day HTS	Informing households on the benefits and availability of testing for HIV; scheduling household level testing appointments and/or actively recruiting clients for same day HTS
3. Documentation	Questionnaire and database	Questionnaire interview on: 1. Personal Data 2. Demographic characteristics 3. HIV testing history 4. HIV test results 5. HIV prevention and risk 6. TB/STI screening 7. Referral 8. Linkage to care 9. (bi) monthly, quarterly, annual reports	Questionnaire interview on: 1. Demographic characteristics 2. HIV testing history 3. HIV test results 4. TB/STI screening 5. HIV prevention and risk 6. TB/STI screening 7. Referral 8. Linkage to care 9. (bi) monthly, quarterly, annual reports
	Payment	Paid per normal payroll	Monthly payment claims to franchisor
4. Basic package of services	Pre-test	1. Information and education 2. Screening 3. Condom education and distribution	1. Information and education 2. Screening 3. Condom education and distribution
	Test	1. Informed written consent 2. HIV testing using national algorithm	1. Informed written consent 2. HIV testing using national algorithm
	Post-test	1. tailor-made counselling based on test result(s) and life situation 2. referral for psychosocial support 3. referral for HIV treatment and care 4. development of tailored linkage plans and timelines looking at individual barriers and concerns 5. if negative: risk reduction counselling, appropriate HIV prevention services	1. tailor-made counselling based on test result(s) and life situation 2. referral for psychosocial support 3. referral for HIV treatment and care 4. development of tailored linkage plans and timelines looking at individual barriers and concerns 5. if negative: risk reduction counselling, appropriate HIV prevention services
5. Follow up	Referral to HIV care and treatment	Discuss referral health facility	Discuss referral health facility
	Linkage and service update	Actively follow up to verify and document linkage to care	Actively follow up to verify and document linkage to care

### Setting

The study will be conducted in the designated project areas with high population densities (between 3200 and 7400 people square kilometre compared to district average of 1110 people per square kilometres) (http://worldpopulationreview.com/world-cities/pretoria-population/), low income, rural, urban and peri-urban settlements in Tshwane metropolitan city, South Africa’s administrative capital. Tshwane district is resident to 3.3 million people with young people (15–34 years) making up 37% of the total population (Mid-Year Population Estimates 2016, Stats SA). An estimated 24.3% live in poverty (http://www.statssa.gov.za). Tshwane’s HIV epidemic requires attention and it is characterised by (i) a low HIV testing coverage of 21.6% which is below national average (23%), (ii) a 2.4% increase in the proportion of people living with HIV from 2016 to 2017; (iii) a third highest attrition rate (out of all districts of South Africa) of people on antiretroviral therapy that is well below the national average of 58.9% and low male condom distribution [28]. The study will be conducted in three of the seven sub districts in the Tshwane metropolitan city. These sub-districts were purposively selected because of their higher than average HIV prevalence and population densities. A significant population lives in dense housing, with poor access to electricity and water. These sub districts have a predominantly black African population. In terms of health, Tshwane district is among the 27 priority districts, referred to as high HIV burden districts, with high HIV incidence and prevalence within South Africa’s 52 districts.

### Participant inclusion criteria

In each study arm any person aged 12 months or older, living in the study community and not having tested for HIV in the last 6 weeks will be offered HIV testing.

### Participant recruitment, consenting and retention

Each study arm conducts participant mobilisation to enable HIV testing. Prior to HIV testing, FPD counsellors and SF counsellors will undertake some community mobilisation /demand creation activities through its dedicated personnel. Councillors in each study arm will visit homes in the community and offer HIV testing to each available participant using the inclusion criteria described above. The consenting process will allow collecting contact details including those of next of kin and name of preferred referral clinic for linkage to care follow up. The baseline interview will be conducted soon after a participant signs the consent form while end line data will be collected during participant follow up as described next.

### Follow up

The purpose of participant follow-up in each arm is to collect linkage to care information from clients who tested HIV positive which include whether one is linked to care or not, date linked to care, result of linkage to care, services received (including initiation on antiretroviral treatment) and challenges to linkage to care and treatment, if any. Counsellors will extract and maintain a log of clients’ date of expected follow up – 6 weeks post-testing, clinic name, date of test and identification details including name, gender, age and national registration identity number. Counsellors will visit all referral clinics in the study community and collect these details which are entered into HIV testing registers. The information will be entered onto referral forms issued to the HIV positive clients during post-counselling and entered into the database. Those not yet linked to care will be called telephonically and or visited to encourage them to link with health facilities for treatment. A maximum of six attempts will be made to encourage participants to link with care before they are regarded as lost to follow up. The follow up process will be done within a period of six months post testing.

### Sample size and sampling

Based on current rates of the CBCT programme, which were used to come up with targets for the SF model, data will be analysed after 12 months of programme implementation. Each arm will have 19 counsellors recruiting at least 100 clients per counsellor per month for 12 months excluding weekends and holidays. Requiring counsellors to test 100 clients per per month is a conservative assumption that considers SFs as new players in conducting CBCT to emphasize accuracy and quality in CBCT work. Based on this assumption and calculation, a total of 91,200 participants are expected to be recruited in 24 months and half of these (45,600) will be recruited by SFs while the other half by FPD. A 12-month follow up and a final 24 months evaluation to assess programme effectiveness will be conducted. No randomisation of counsellors or clients will be done. All sub districts were selected based on current rates of HIV infection, which is highest in the townships where predominantly black Africans reside.

### Control arm - FPD HTS

In order to identify people infected with HIV (PLHIV) and bring them into HIV care, FPD designed and implemented a CBCT programme in 2013 that ran through 2018 [29]. The programme aimed to make significant inroads towards the UNAIDS’s 90–90-90 goal [30] through the alignment with the South African Government’s (SAGs) National Strategic Plan on HIV, STIs and TB (NSP 2012–2016) [31] and the PEPFAR/SA HIV Prevention Strategy [32]. To implement the CBCT strategy, FPD formally advertised and employed counsellors to conduct HTS. The counsellors attended a 10-day training programme covering HTS and linkage to HIV care including documentation, approaching communities, consenting as shown on Table 2. FPD managed counsellors’ daily HTS activities and remunerated them monthly without necessarily pegging the salary to HTS outputs. The client recruitment strategy for testing is described as home-based in which counsellors moved door-to-door recruiting clients in the community. FPD teams, who include a driver/counsellor and at least four other counsellors per team, drive from the office to the communities in the selected study/project sites daily to conduct HIV testing and travel back to the office. Counsellors will test and refer clients for linkage to care. Linkage to care will be confirmed by checking clinic registers and Tier.net, an electronic patient management system used for capturing patient-level data on HTS, pre-ART and ART services. Counsellors will follow up clients for up to six months to ensure that they are linked to care.

### Intervention arm - SF HTS or SFs

In 2016, FPD CBCT introduced a separate Social franchise testing model [27] into the existing CBCT approaches (See Table 2). The programme aims at assessing effectiveness of a community-based HTS study implemented through social franchisees in Tshwane district in Gauteng province, South Africa. The model aims at providing implementation evidence by assessing success or failure of the model to inform roll out through a public-private partnership between government and non-governmental organisations. As part of the recruitment process, FPD advertised and encouraged individuals and small-scale business people, either employed or unemployed, to apply for the SF opportunity. Applicants will be interviewed by an FPD recruitment and selection panel. Successful SFs will be provided with a 10-day training on HTS, documentation, referral for ART and business management. The training on small business management is aimed at ensuring SFs obtain skills in management of mall-scale business including CBCT resources, ordering and disbursing CBCT materials and ledger balancing. Each SF (*n* = 19) will sign a contract with FPD to deliver a standards-based product of CBCT (HIV testing and linkage to care package) to decide where to conduct HIV testing daily in the designated communities, decide demographics of people to target daily, mobilise communities, families and individuals regularly, recruit clients, test them, and to link HIV positive clients to referral health facilities within the communities per agreement with clients. SFs will deliver a common brand that FPD implements and systematizes. The role of the franchisor, FPD, will be to prescribe HTS standards, recruit, train and accredit SFs, including deregistering SFs that fail to meet prescribed CBCT brand/standards. FPD will also maintain quality assurance of the SFs through making announced and unannounced visits to the SF offices and areas of work. In addition, FPD will support the SFs by establishing linkages between SFs and the referral health facilities where the SFs will refer clients for HIV prevention, care and treatment. Furthermore, FPD will facilitate access to HTS commodities and waste disposal services, verification of linkage to HIV care and treatment, access to information, education and provide communication materials for HIV prevention and marketing as well as paying SFs in accordance with numbers tested and linked to care and treatment.

SFs will be remunerated per specific measurable work activities done in order to track progress and measure costing of the project, while also delivering agreed outputs and maintaining the brand. As such, R40^1^ will be paid per HIV test conducted. This amount remunerates a basic package of HTS services to each client tested that includes consenting a client, conducting an HIV test, distributing condoms, providing client tailor made HIV prevention messages and relevant referrals,^2^ screening for TB and STI and subsequently making necessary referrals, documenting the client. A second, and much bigger remuneration, of R100 will be paid for every HIV positive client linked to HIV care and treatment programmes in the health system. This larger amount is aimed at motivating linkage to care which proved challenging for most counsellors implementing CBCT programmes in the country [33, 34]. SFs will be contracted to test a minimum of 100 clients per month. This minimum requirement was determined by calculating the average time required to conduct HIV testing of an average of 5 people per day while allocating time to conduct other duties including mobilisation, documentation and business management aspects. In order to motivate the SFs to study communities to identify and target HIV hotspots [35, 36], it is a contractual requirement to find at least 5 HIV positive individuals in the 100 people tested. This translates to a minimum 5% HIV positivity target in Tshwane district. The target for linkage to care was at least 4 (80%) of the 5 HIV positive clients being linked to care. Consideration was made of the challenges of linkage to care based on FPD’s experience of running the CBCT programme. The project will review the targets upwards once SFs demonstrated delivery of adequate and quality work in order to align to the UN 90–90-90 target for South Africa to link 90% of the HIV positive people identified [30, 37]. Regular (weekly, bi-monthly, monthly, and quarterly) reports, field visits and observations will be made by FPD to monitor program performance and address any challenges. FPD will audit the SFs’ work and provide regular feedback to SFs to assess their progress. The same linkage to care process as in the FPD HTS will be followed by counsellors.

### Outcomes

The study has four primary outcomes for programme success: 1) *HIV testing uptake rate,* is defined as all clients who received a basic package of HTS, including HIV testing, divided by the total number of clients offered HIV testing multiplied by 100, 2) *HIV positivity* is defined as the number of clients testing HIV positive divided by the total number of clients tested for HIV multiplied by 100, 3) *Linkage to HIV care and treatment rate* is a proportion of all clients tested positive during the current reporting period and successfully linked to care, divided by the number of all HIV positive clients referred for linkage during the current reporting period multiplied by 100. 4) *Average Counsellors’ remuneration cost* per person tested, per HIV positive test and per person linked to care will be calculated to determine the average cost of employing counsellors in each study group per outcome described above. Secondary outcomes include 5) *App use rate,* is the total number of participants whose data were primarily captured on the app divided by the total number of clients tested multiplied by 100, 6) *First time HIV testers rate* is the proportion of persons identified as testing for HIV for the first time divided by the total number of people tested in a given period multiplied by 100, 8) *Couples HIV testing rate* is defined as the proportion of two people, identifying themselves as partners, testing and receiving results together divided by the total number of clients testing multiplied by 100.

### Data collection: use of an electronic mobile application (the app)

**Fig. 1:**
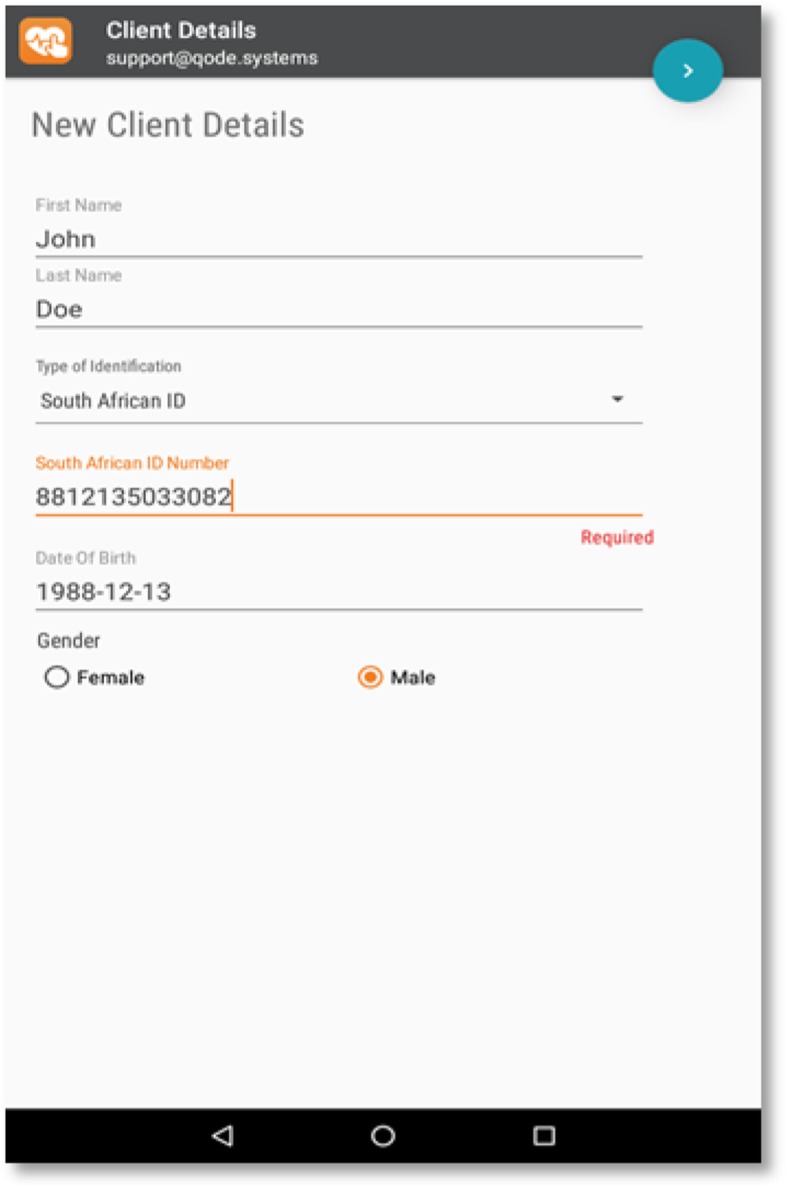
Picture showing the Mobile App data entry form designed for this study

One of the secondary objectives of the study is to test the effectiveness of a mobile electronic data collection application (the App) in both study groups to collect real time quality data required to assess programme effectiveness and to strengthen linkage to care services. Data will be collected using the App, as well as paper-based tools developed by FPD. The App was designed for this study. The use of the App was deemed to ease the process of linkage to care as client data will be accessible on the South African Government electronic databases and could be accessed by others on the CBCT programme in real time using the Qode Insight system with which to track individual clients’ linkage to care. If a client is linked, the system will capture the name of health facility where a client is linked to care and date of linkage and initiation of care, among other details, through a password-protected online reporting portal. Clients who are lost to follow-up in the treatment cascade will be identified through the Qode Insight system. They will be tracked, and if found, counselled with the aim to reinitiate them on treatment at health facilities. In addition, data captured on the App minimises data entry challenges of accuracy and completeness. Capturing data on paper means that it must be recaptured onto the electronic database support systems (the Bulk Online Capture System or BOCS) that allows the capture of data in situations where the mobile application was not used. The use of the App is therefore expected to contribute towards the success of the entire CBCT programme. All counsellors will be trained for at least one day on using the app. The app has a minimum of 54 variables per participant while a client who tested HIV positive has at least 22 additional variables to complete and those referred for linkage to care will have at least 20 more variables to complete. The app will be completed by the counsellors who administer the interviews. The completion of the app will take place throughout the counselling and testing and linkage process. Every indicator data entry is captured directly onto the application. Participants will be interviewed in vernacular. The app has relevant skip patterns and prompts to suit participants’ various situations and ensure that all relevant questions are entered. The database is managed at FPD by a database developer. Figure 1 shows a picture of a tablet app where client data is entered.

The app will be accessible through using an assigned password to each counsellor. Each counsellor will have a user name/identity (ID) code which reflects in the database to assist programme managers to track progress per counsellor, per day. Data will be captured and saved automatically onto the app and in real-time transferred to the cloud-based database using an internet connection. In cases of limited or no internet connection, the app saves an encrypted copy of the data on the tablet/device and the counsellor then uploads the data manually, through the push of a single button, onto the database when the internet connection becomes available. Counsellors conduct manual uploads at the end of each working day to ensure all data are uploaded onto the database. After development, the App was tested and data were captured, retrieved, analysed and compared for completeness, validity and accuracy.

### Measures

HIV testing and counselling will be conducted following the South African algorithm [38]. An HIV positive status will be assessed by a first test using Alere Determine HIV-1/2 Rapid Test (Alere Medical Co. Ltd., Matsudo-Shi Chiba, Japan) which, if positive, will be confirmed by Abon HIV 1/2/0 Tri-Line Rapid Test (Abon Biopharm, Hangzhou, R. R China). In case of discordant results, final results will be confirmed by Western Blot. Data collected to facilitate linkage to care include a participant’s national ID number, age, gender, place of residence, testing place name, sub district, two telephone/mobile phone numbers, referral clinic name, and client register number. In couples testing, identifying personal data will be collected to assess discordant results. Participants will be asked if they identify themselves as belonging to any key population groups including female sex workers (FSWs), men who have sex with men (MSM), injection drug users (IDUs), and if they are clients of sex workers.

The tool also collects data on participants’ HIV testing history – if they ever tested for HIV before or they were first time testers. In terms of HIV prevention, the questionnaire asks if the participants received condoms and if they were referred for voluntary male medical circumcision, antenatal care, gender-based violence care, TB care, family planning care, antiretroviral treatment or other social services. For TB screening, a tool with six questions will be administered to assess if a participant ever, for longer than two weeks, coughed up blood in sputum, had sudden and unexplained weight loss, night sweats, fever or been in close contact with someone coughing for more than two weeks [39]. A typical question is: “Have you been coughing/coughed for longer than two weeks? Participants were requested to respond affirmatively with a “Yes” or otherwise with a “No”. To screen for sexually transmitted infections (STIs), four questions will be asked to assess if a participant ever had unprotected sex with an HIV or STI suspect, abnormal or smelly discharge or bleeding from a vagina or penis, or experienced pain during sex or when passing urine. A typical question is: “Did/Do you have an abnormal or smelly discharge from the vagina or penis?” Participants will be requested to choose yes or no as answers to the question. Female participants will be screened for pregnancy while male participants will be asked if they were circumcised. A screening tool for non-communicable diseases assesses levels of blood sugar and blood pressure.

### Ethical considerations

Written informed consent will be sought from each potential participant. In keeping with the relevant South African Law including the Children’s Act, and the South African Good Clinical Practice Guidelines (2006), all children between 12 and 17 can consent to HIV testing without consulting a parent or guardian [40, 41]. For those under 12, parental consent will be sought and when provided, assent will be sought from the child. Participants will be informed that their personal information, demographic, and testing details will be shared with health personnel at referral health facilities to facilitate linkage to care but that no additional people outside the study will be authorised to access participant data without the permission of the Principal Investigator. The benefits of participating in the study include knowing own HIV status, getting a wide range of free health screening including TB, STI, blood sugar and pressure as well as facilitated and accelerated referral to care including ART for those screening/testing positive. However, getting to know HIV status may cause one to feel anxious and/or nervous which is manageable though due to the shorter time it takes to conduct an HIV rapid test and also counselling that highlights the freely available life prolonging medicines for those testing positive. Starting ART earlier is beneficial as proved in a number of studies [42]. The study was approved by the FPD Research Ethics Committee (Protocol 13–2017) and was registered with the Pan African Clinical Trial Registry (PACTR201809873079121).

### Data analysis

Data will be downloaded from the database and retrieved in excel format through an export mechanism and exported to Stata [43] for analysis. The analysis of primary outcomes will be conducted by comparing proportions of HIV testing, positivity rates, linkage to HIV care and treatment rates, app use rates and per counsellor cost by gender and age group in the two study groups. Descriptive statistics will be performed to describe the data. Proportions stratified by age group and gender will be calculated and presented. In comparing proportions between FPD and SF, 95% confidence intervals will be calculated and presented. All analyses will be conducted using STATA 14.0. A costing analysis will be done to assess programme cost per counsellor per HIV test, positivity and linkage to care.

## Discussion

The study seeks to test the efficacy of the SF-delivered CBCT programme to increase HIV testing coverage and linkage to care. To our knowledge, this is the first community-based HIV testing and counselling study to contract social franchisees for HIV testing and linkage with the goal to increase HIV testing and treatment in the population. We believe that the results will be informative not only in Africa, but beyond. If found to be efficacious, the study may have a crucial influence on policy and implementation of CBCT as a public-private sector response to HIV prevention. Findings may also contribute towards an evidence base for community health workers’ contribution in relieving the health burden. The intervention may be an effective implementation tool for reaching first time testers and the hard to reach populations at high risk of HIV infection including men, young people and members of the key populations such as MSM, FSWs, and IDUs. This is because the SFs, who are recruited from and deployed to work in the same community have a better understanding of the local HIV hotspots that they then targeted with HIV testing. In addition, the intervention’s data App may influence the way CBCT programmes can be run with respect to the health information system. This is because the App facilitates effective use of personal health data. Lastly, the intervention may influence the way CBCT and similar health projects should be conducted while strengthening grassroots community health workers, unemployed and self-employed people as social franchisees. In a country where unemployment has reached 27% the training and employment of unemployed local people for SF-CBCT creates better life opportunities.

Our study design has some strengths and limitations. The strengths of the design include that it compares a new model of SFs with existing CBCT approach that has been implemented for over a decade now [14]. That the CBCT approach has been successfully implemented in at least three years offers a good standard of care to compare a new implementation programme, SF, with. The site of the implementation of the study, Tshwane district in Gauteng province, was a good selection for two main reasons. Firstly, Gauteng has an HIV prevalence similar to the national average rate. Secondly, the study sites consisted of rural, urban and peri-urban centres which is comparable to the South African population at large. In addition, the sites also contained heterogeneous populations including foreigners, local indigenous people as well as internal migrants from across other provinces. This population heterogeneity favours the generalisation of the study findings to the greater South African population for the purpose of HIV prevention through testing and treatment. Also, that the population is predominantly black African mirrors the populations that bears the brunt of the HIV pandemic in the country.

However, the fact that the two models will be implemented in the same communities (not cluster based) and that communities and clients are not randomised is a limitation for the study. Only an analysis by individual client level is possible due to non-randomisation. There may be challenges in conducting an economic analysis of costs as getting appropriate estimates of travel in the sub districts as both FPD and SFs counsellors conduct testing in the same sub-districts. On the other hand, not restricting counsellors to specific areas in the study area also enable counsellors to assess hotspots and testing in the areas of choice each day.

We anticipate challenges in linkage to care among the SF CBCT. This is because as independent businesses or self-employed, though using an FPD brand, they may not be as well accepted at health facilities as FPD employees would. Although clients usually live within a walkable distance to the local health facilities, some clients may face challenges in accessing these facilities due to shortage of transport money or experiencing stigma making them unable to visit the facility or choosing to visit facilities far away from their locality to minimise stigmatising situations. Training of CHWs will address some of these challenges. In rare circumstances, clients may be offered transport, when available, to access health facilities. The monitoring and management staff will be available to provide support and resolve any conflicts that the SFs face during referral and linkage to care processes. In addition, regular contact with the franchisor through reporting of data, challenges, financial, and commodity fill up visits will enable any challenges to be addressed during project implementation. The use of the App on tablets in a community, sometimes rural or peri-urban communities, raises challenges of internet connection and or gadget battery flat outs or insecurity/theft in high crime areas leading to completing data on paper and later capturing onto the electronic database with possible challenges related to data completeness and real time availability.

The trial will inform on the importance of health information system including gathering, processing, sharing and storage. The success of linkage to care is also hinged on collecting reliable identifying, tracking and locating information. A relationship of trust, which is governed by the consenting process, helps to obtain such private information [44] for follow-up purposes. As observed in large health information systems in South Africa, missing data is a challenge [45, 23]. In our study, missing data may be a result of the interviewer not strictly observing prompts or personnel capturing data from paper-based forms missing some values while capturing. Refresher training and close monitoring of the project will minimise missing data. One of the strengths of the app is that it has prompts that require the counsellor to respond to compulsory questions before proceeding to the next questions.

We also anticipate challenges in data accuracy, completeness, quality assurance and verification of testing of clients. Similar challenges have been reported in an evaluation of social franchisees in the health provision in developing countries [46]. Nevertheless, our monitoring system through site visits, data verification and auditing process, and integrating databases with the national department of health to facilitate linkage to care will, among other things, help to deal with such challenges.

In summary, the trial seeks to recruit, train and equip unemployed, self-employed and employed community members to increase community-based HIV testing and treatment. The programme data will help to generate evidence of success or failure of the SF CBCT approach based on the programme outcomes including HIV uptake, positivity, linkage to care rates and remuneration cost per test. The trial is unique in that it uses the social franchise approach to increase coverage of HIV testing and treatment and at the same time creating employment opportunities for unemployed people and provides free training and support in business management. The SFs will receive skills that they will use beyond the project life span. The study results will inform the Franchisor of potential limitations to the programme and ways to circumvent those challenges going forward. The results will also inform the South African Department of Health and the funding organisations on new and effective approaches to increase HIV testing and treatment including identifying at high-risk populations and the hard to reach population with HIV testing. The results are awaited by key stakeholders including the donors, policy makers and researchers.

## Data Availability

Data sharing is not applicable to this article as no datasets were generated or analysed during the current study.

## Abbreviations

ART: Anti-retroviral therapy
CBCT: Community-based HIV counselling and testing
CHW: Community health workers
FPD: Foundation for Professional Development
HTS: HIV testing services
PEPFAR: President’s Emergency Plan for AIDS Relief
SA: South Africa
SF: Social Franchise(e)
STI: Sexually transmitted infection
TB: Tuberculosis

## Footnotes

1 Exchange rate was 1 Unites States Dollar (US$) = 12.50 South African Rand

2 Referrals were tailor-made based on HIV risk assessment/profiling per client during the pre-and post-test counselling sessions
